# Integrated Transcriptomic–Metabolomic Analysis Reveals the Effect of Different Light Intensities on Ovarian Development in Chickens

**DOI:** 10.3390/ijms25168704

**Published:** 2024-08-09

**Authors:** Xiaoli Zhou, Yuhang Xu, Cheng Fang, Chutian Ye, Weiming Liang, Zhexia Fan, Xuerong Ma, Aijun Liu, Xiquan Zhang, Qingbin Luo

**Affiliations:** 1 College of Animal Science, South China Agricultural University, Guangzhou 510642, China; 2 State Key Laboratory of Livestock and Poultry Breeding, South China Agricultural University, Guangzhou 510642, China

**Keywords:** light intensity, ovarian development, transcriptomics, metabolomics, partridge chicken

## Abstract

Light is a key environmental factor regulating reproduction in avians. However, the mechanism of light intensity regulating ovarian development is still unclear. In this study, 5-week-old (5 wk) partridge broiler breeders were randomly divided into a low-light-intensity group (LL group) and a natural-light-intensity group (NL group) (*n* = 100). In the rearing period (5 wk to 22 wk), the light intensity of the LL group and NL group were 0.41 ± 0.05 lux and 45.39 ± 1.09 lux, and in the laying period (23 wk to 32 wk) they were 23.92 ± 0.06 lux and 66.93 ± 0.76 lux, respectively. Samples were collected on 22 wk and 32 wk. The results showed that the LL group had a later age at first egg and a longer laying period than the NL group. Serum P4 and LH levels in the LL group were higher than in the NL group on 22 wk (*p* < 0.05). On 32 wk, P4, E2, LH and FSH levels in the LL group were lower than in the NL group (*p* < 0.05). Ovarian transcriptomics and metabolomics identified 128 differentially expressed genes (DEGs) and 467 differential metabolites (DMs) on 22 wk; 155 DEGs and 531 DMs on 32 wk between two groups. An enrichment analysis of these DEGs and DMs identified key signaling pathways, including steroid hormone biosynthesis, neuroactive ligand-receptor interaction. In these pathways, genes such as *CYP21A1*, *SSTR2*, and *NPY* may regulate the synthesis of metabolites, including tryptamine, triglycerides, and phenylalanine. These genes and metabolites may play a dominant role in the light-intensity regulation of ovarian development and laying performance in broiler breeders.

## 1. Introduction

Light is a key environmental factor influencing many animals’ physiological processes and behaviors [1]. In avian species, improved light conditions not only promote the secretion of hormones related to growth, maturation and reproduction, but also enable avians to establish biological rhythms synchronized with physiology [2,3,4,5].

Light consists of three main factors: light color, light intensity and photoperiod (day length). It has been demonstrated that light is an important regulator of reproduction in avians [6,7]. For instance, when changing the light conditions, such as the photoperiod, avians can receive light signals and regulate ovarian development through the hypothalamic–pituitary–gonadal (HPG) axis. During this process, the light signal is transferred to neuroendocrine signals, which can regulate the secretion of gonadotropins, such as follicle-stimulating hormone (FSH) and luteinizing hormone (LH). Subsequently, gonadotropins regulate ovarian development by influencing the ovarian secretion of steroid hormones such as estrogen (E2) and progesterone (P4) through blood circulation, and well-developed ovaries can ensure the high-laying performance of avians [8,9].

Though there are many studies on the mechanisms of the light-based regulation of reproduction, most of them focus on photoperiod [3,10,11,12]. Studies have also found that light intensity also plays an important role in avian ovarian development. Studies in hens found that high- (121.8 lux) and medium-light-intensity (57.4 lux) treatment had higher egg laying than low-light-intensity (11.9 lux) treatment [13]. In Pekin ducks, the increase in light intensity promoted reproductive performance [14]. In Shan-partridge ducks, the 900 lux group had a higher laying rate than the 600 lux group under a 12 L:12 D photoperiod [15]. Those studies have demonstrated that light intensity can affect laying performance in poultry. However, the mechanism of light intensity regulating avian ovarian development is still unclear.

Partridge chicken is a dual-purpose chicken for meat and eggs and has a significant position in the Chinese poultry industry. Improving the breeding performance of partridge broiler breeder hens has important economic benefits. In the practical production of breeder hens, light management will directly affect egg production. However, no standard regulations of light intensity have been established for partridge chicken. Early photostimulation has been shown to reduce reproductive performance in broiler hens [16]. In current commercial production in China, partridge chickens are still raised in semi-open housing and receive natural light. This natural light intensity is sufficient to induce ovarian development, which will result in decreased laying performance by receiving early photostimulation. Therefore, based on this rearing system, we shaded the chickens’ house with black cloth and kept the chickens in a low-light-intensity environment during the growing and laying period. This study aims to explore whether this protocol can prolong the egg-laying period and enhance reproductive performance in broilers, and reveal the mechanism of light intensity regulation on avian ovarian development.

Sequencing technology has evolved, allowing transcriptomic and metabolomic technologies to have broader use. Transcriptomics can reveal the changes in gene expression patterns, and metabolomics can reveal the changes in metabolite profiles. The analysis and integration of these two technologies can provide a more comprehensive understanding of biological systems, allowing for a deeper exploration of their functions and potential regulatory mechanisms [17,18]. In this study, we integrated transcriptomic and metabolomic analysis to investigate the effects of light intensity on ovarian development and reproductive performance in chickens, providing a theoretical basis for the management of light intensity in the production of partridge chicken.

## 2. Results

### 2.1. Effect of Different Light Intensities on Laying Performance

**Figure 1:**
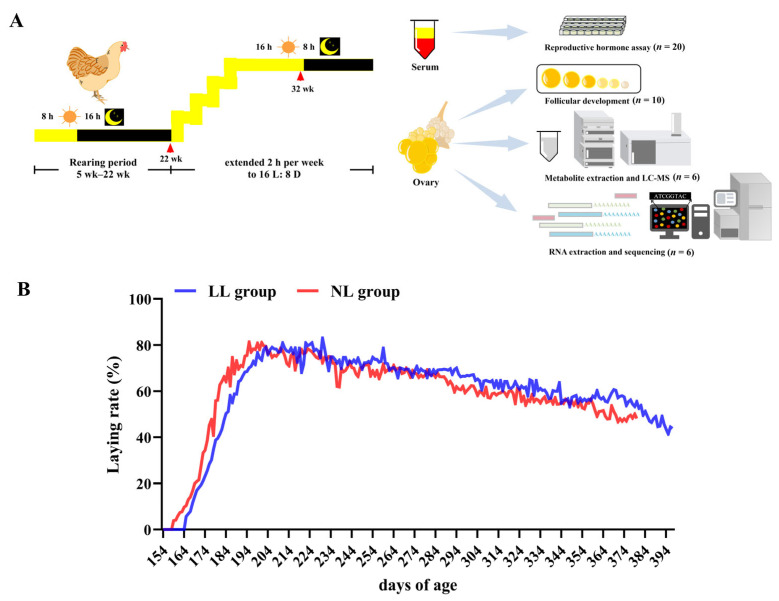
Effect of different light intensities on laying performance. (**A**) Experimental design of this study. Two hundred 5-week-old (5 wk) partridge broiler breeders with similar body weights were selected and randomly divided into low-light-intensity group (LL group) and natural-light-intensity group (NL group) (*n* = 100). The light intensity of the LL group and NL group from 5 wk to 22 wk were 0.41 ± 0.05 lux and 45.39 ± 1.09 lux, respectively; and from 23 wk to 32 wk, they were 23.92 ± 0.06 lux and 66.93 ± 0.76 lux, respectively. Tissue and blood samples were collected on 22 wk and 32 wk. (**B**) Laying rate.

In order to elucidate the regulatory mechanism of light intensity on ovarian development, we first investigated the effects of different light intensities on egg production performance. As shown in Figure 1B and Table 1, the laying performance of the low-light-intensity group (LL group) was better than the natural-light-intensity group (NL group). The age at a 5% laying rate and the age at the laying peak of the LL group was significantly later than the NL group (*p* < 0.05), but the total days of laying and the days of laying rate > 70% of the LL group were longer than that of the NL group (*p* < 0.01). The total laying rate of the LL group was higher than the NL group.

### 2.2. Effect of Different Light Intensities on Ovarian Development

**Figure 2:**
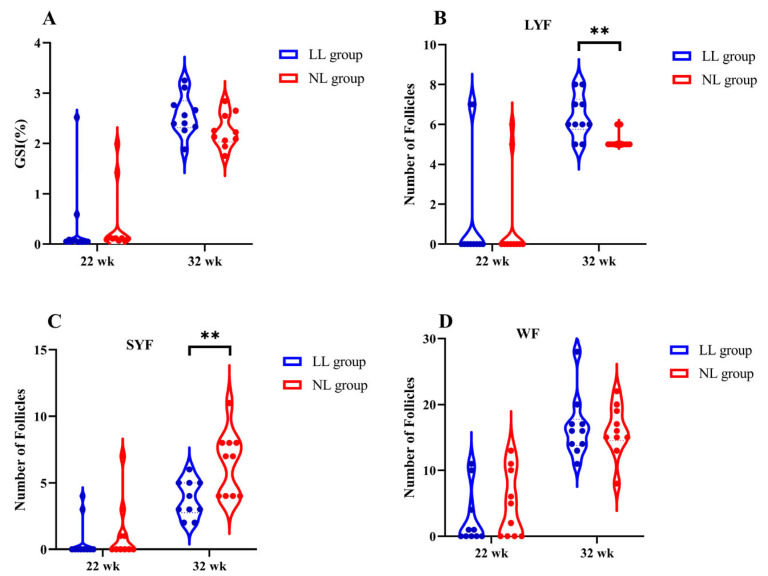
Effects of different light intensities on GSI% and numbers of follicles. (**A**) GSI% (GSI% = gonadal weight/body weight × 100%); (**B**) Number of large yellow follicles (LYFs, follicle diameter > 8 mm); (**C**) Number of small yellow follicles (SYFs, follicle diameter = 6–8 mm); (**D**) Number of white follicles (WFs, follicle diameter < 6 mm). Data are presented as the mean ± SEM, *n* = 10. ** indicates *p* < 0.01.

Subsequently, we calculated the gonadosomatic index (GSI% = gonadal weight/body weight × 100%) and counted the follicle number to explore the effect of different light intensities on ovarian development. As shown in Figure 2, the GSI%, numbers of large yellow follicles (LYFs, diameter > 8 mm), small yellow follicles (SYFs, diameter = 6–8 mm) and white follicles (WFs, diameter < 6 mm) were no different between the LL group and NL group on 22 wk. On 32 wk, the number of LYFs in the LL group was higher than in the NL group (*p* < 0.01). The number of SYFs in the LL group was significantly lower than in the NL group (*p* < 0.01). GSI% and the number of WFs in the LL group were also higher than that of the NL group, but there was no significantifference between the two groups.

### 2.3. Effect of Different Light Intensities on Reproductive Hormone Levels

Ovarian development is regulated by reproductive hormones. We further investigated the effect of different light intensities on reproductive hormone secretion. As shown in Table 2, the serum hormone levels of P4, E2, LH and FSH were measured. The P4 and LH levels in the LL group were significantly higher than that of the NL group on 22 wk (*p* < 0.05). The E2 and FSH levels in the LL group were also higher than in the NL group on 22 wk, but there were no significant differences between the two groups (*p* > 0.05). On 32 wk (Table 3), results showed that the levels of P4, E2, LH and FSH were significantly lower in the LL group than in the NL group (*p* < 0.05).

### 2.4. Transcriptomics Data Summary

We obtained a total of 116.74 million clean reads from RNA transcripts after filtering. The Q20 (percentage of reads with a Phred quality value > 20), Q30 (percentage of reads with a Phred quality value > 30) and the GC content of the clean reads ranged from 97.61 to 99.39%, 93.42–97.33% and 47.18–49.19%, respectively. The mapping rate of the 24 samples ranged from 94.05 to 95.59% (Table S1).

### 2.5. Identification of DEGs and Functional Analysis

**Figure 3:**
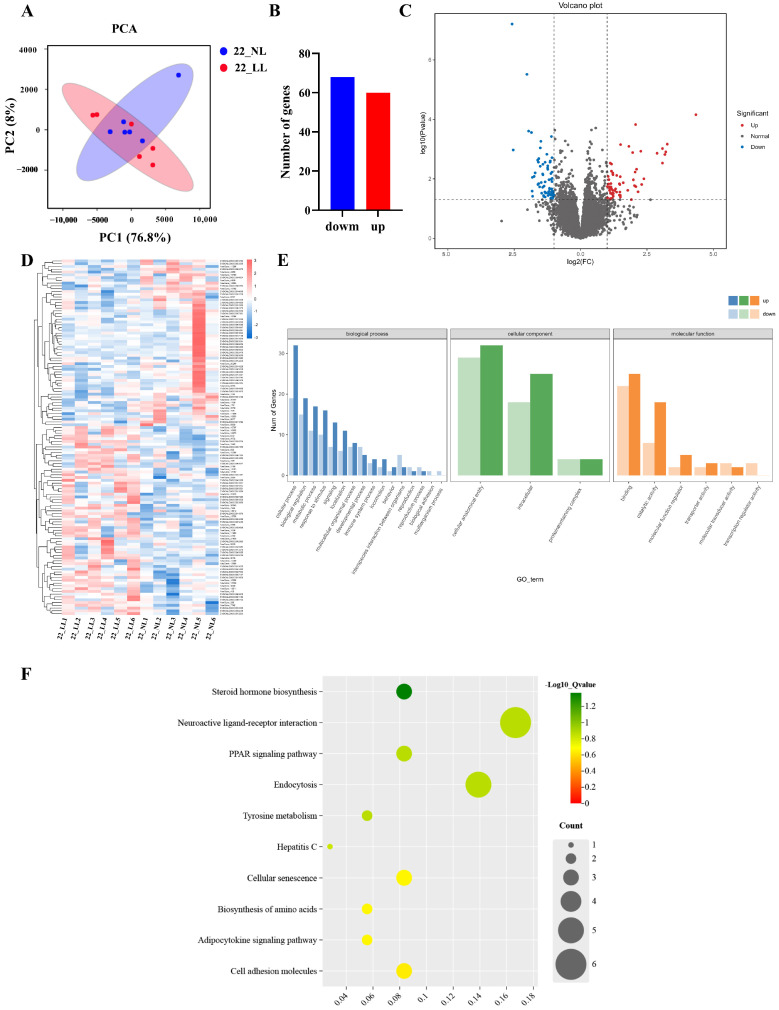
Transcriptomic analysis in ovaries under different light intensities on 22 wk. (**A**) PCA plot; (**B**) DEGs bar plot; (**C**) Gene volcano plot; (**D**) DEGs clustering heatmap; (**E**) DEGs GO enrichment analysis plot; (**F**) DEGs KEGG enrichment analysis plot.

To investigate the effect of different light intensities on gene expression, the transcriptome data were compared between the NL group and the LL group at different sampling ages. As shown in Figure 3 and Table S2, the comparison showed a total of 128 DEGs (60 up-regulated and 68 down-regulated) between NL group and LL group on 22 wk. Subsequently, GO and KEGG analyses were performed to understand the functional significance of DEGs. In the GO enrichment analysis (Figure 3E and Table S3), we found enrichment in biological processes, such as biological regulation, the metabolic process, reproduction, the cellular process and signaling. Enrichment was discovered also in cellular components, such as the cellular anatomical entity, and the intracellular and protein-containing complex. In molecular functions, DEGs were predominantly enriched in binding, catalytic activity and molecular function regulator. Through KEGG enrichment analysis (Figure 3F; Table S4), we observed that these DEGs were mainly enriched in pathways such as neuroactive ligand–receptor interaction, endocytosis, steroid hormone biosynthesis and the PPAR signaling pathway.

**Figure 4:**
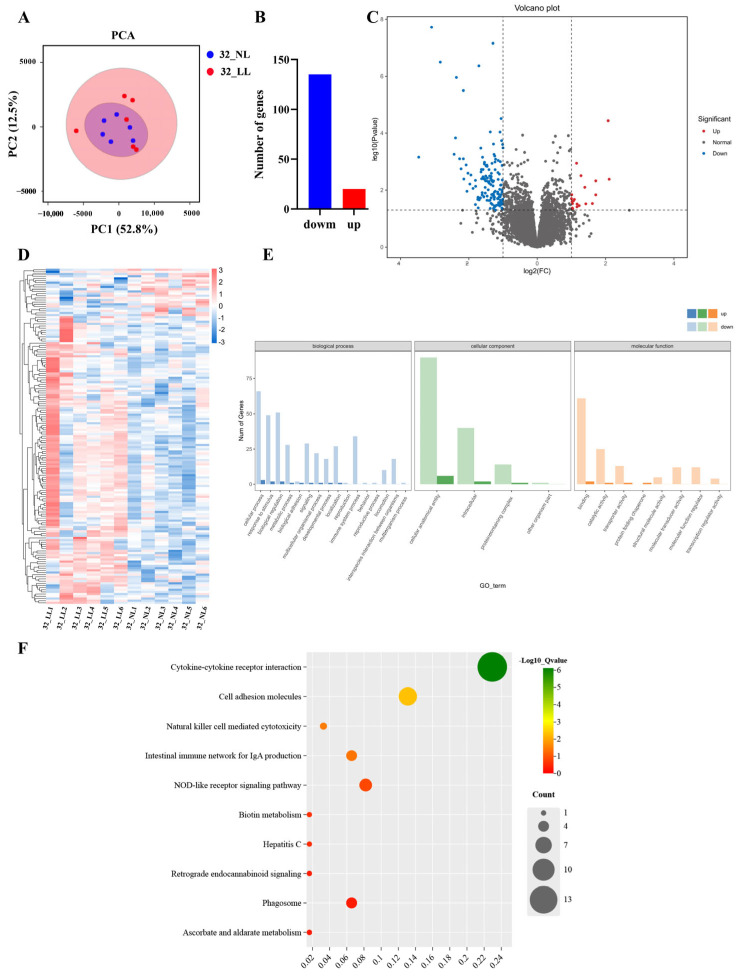
Transcriptomic analysis in ovaries under different light intensities on 32 wk. (**A**) PCA plot; (**B**) DEGs bar plot; (**C**) Gene volcano plot; (**D**) DEGs clustering heatmap; (**E**) DEGs GO enrichment analysis plot; (**F**) DEGs KEGG enrichment analysis plot.

As shown in Figure 4 and Table S5, the comparison showed a total of 155 significantly DEGs (20 up-regulated and 135 down-regulated) between the NL group and the LL group on 32 wk. In the GO enrichment analysis (Figure 4E; Table S6), we found enrichment in biological processes, such as the cellular process, biological regulation, the response to stimulus and the metabolic process. Enrichment was also discovered in cellular components such as the cellular anatomical entity, the intracellular and the protein-containing complex, and other organism parts. In molecular functions, DEGs were predominantly enriched in binding, catalytic activity, transporter activity and molecular function regulator. Through KEGG enrichment analysis (Figure 4F; Table S7), we observed that these DEGs were mainly enriched in pathways such as cytokine–cytokine receptor interaction, cell adhesion molecules, and the NOD-like receptor signaling pathway.

### 2.6. Identification of DMs and Functional Analysis

**Figure 5:**
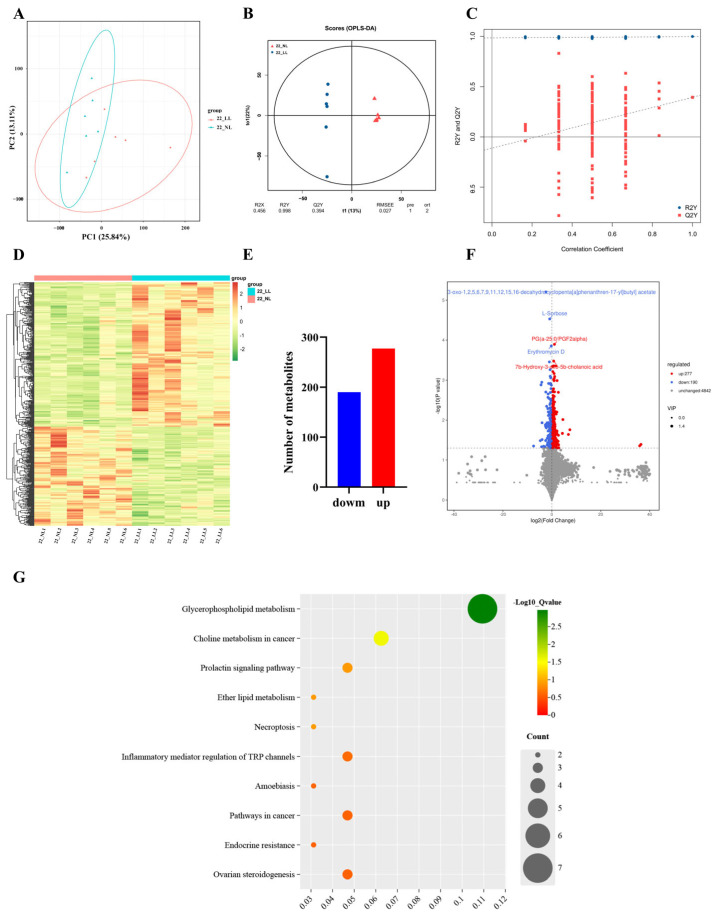
Metabolomic analysis in ovaries under different light intensities on 22 wk. (**A**) PCA plot; (**B**) OPLS-DA scores plot; (**C**) OPLS-DA model validation plot; (**D**) DM clustering heatmap; (**E**) DM bar plot; (**F**) metabolites’ volcano plot; (**G**) DM KEGG enrichment analysis plot.

**Figure 6:**
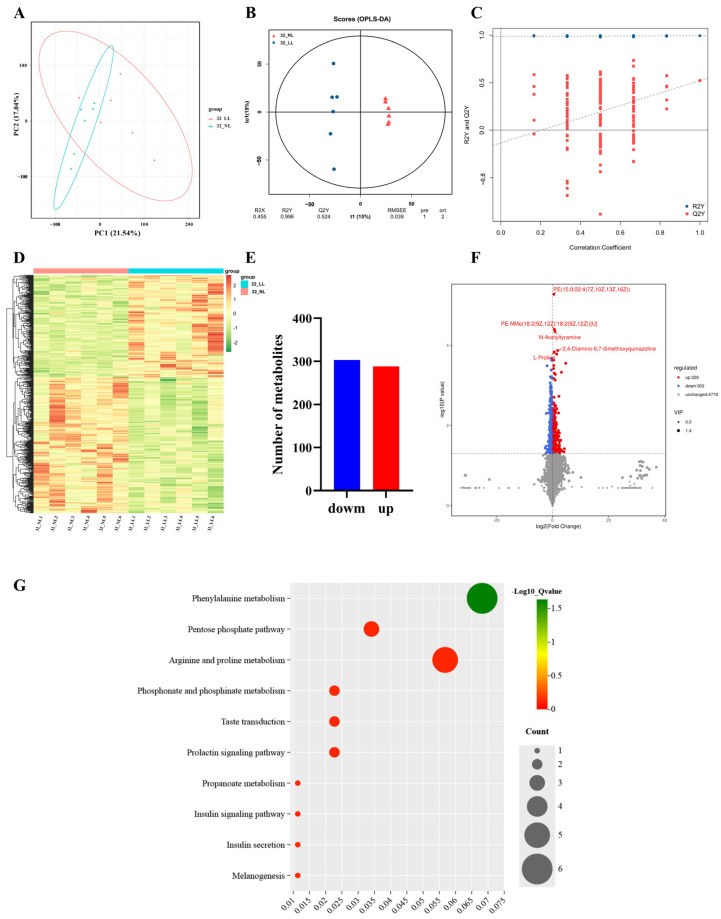
Metabolomic analysis in ovaries under different light intensities on 32 wk. (**A**) PCA plot; (**B**) OPLS-DA scores plot; (**C**) OPLS-DA model validation plot; (**D**) DM clustering heatmap; (**E**) DM bar plot; (**F**) metabolites’ volcano plot; (**G**) DM KEGG enrichment analysis plot.

In this study, metabolomics analysis was performed on ovary samples from the NL group and the LL group to detect overall biochemical changes (*n* = 6). The PCA method was used to observe the overall distribution of metabolites and the difference between samples from NL group and LL group on 22 wk and 32 wk (Figure 5A and Figure 6A). The OPLS-DA (orthogonal projections to latent structures-discriminant analysis) showed significant separation between the groups, and the permutation tests further indicated that the model had a low overfitting risk and good reliability (Figure 5B,C and Figure 6B,C).

A total of 5309 metabolites were identified. As shown in Figure 5D–F and Table S8, the comparison showed a total of 467 DMs (277 up-regulated and 190 down-regulated) between the NL group and LL group on 22 wk. To further investigate the biological functions of DMs, we performed an analysis of those DMs using KEGG enrichment. DMs were mainly enriched in pathways such as glycerophospholipid metabolism, steroid hormone biosynthesis, the prolactin signaling pathway and ovarian steroidogenesis (Figure 5G; Table S9).

As shown in Figure 6D–F and Table S10, the comparison showed a total of 531 DMs (288 up-regulated and 303 down-regulated) between the NL group and LL group on 32 wk. The PCA and heatmap results showed that the samples were clearly clustered by sampling time in terms of metabolite expression patterns. To further investigate the biological functions of DMs, we performed an analysis of those DMs using KEGG enrichment (Figure 6G; Table S11). DMs were mainly enriched in pathways such as phenylalanine metabolism, arginine and proline metabolism, steroid hormone biosynthesis and the prolactin signaling pathway.

### 2.7. Integrative Metabolomics–Transcriptomics Analysis

**Figure 7:**
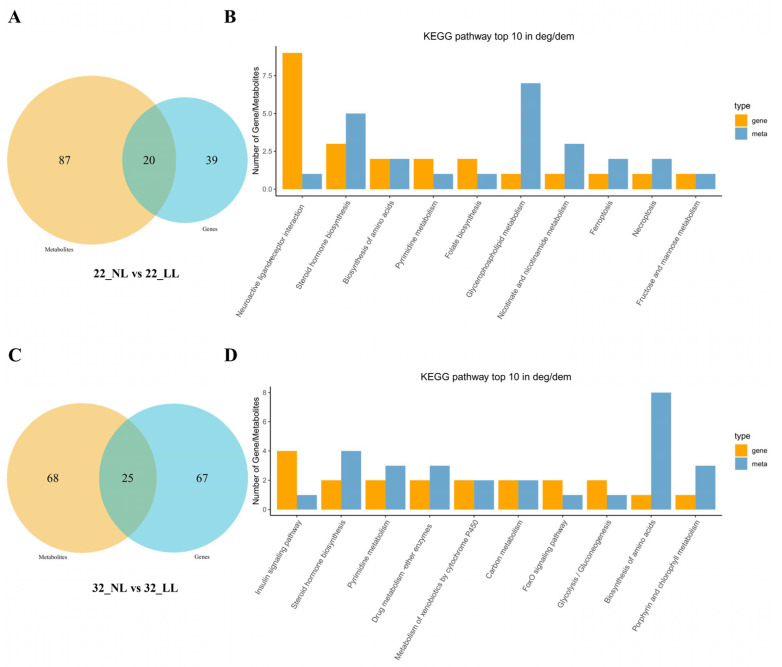
Integrated analysis of transcriptomics and metabolomics in ovaries under different light intensities. (**A**) Venn diagram of intersection of DEG and DM enrichment signaling pathways on 22 wk; (**B**) top 10 pathways of the intersection of DEG and DM enrichment signaling pathways on 22 wk; (**C**) Venn diagram of intersection of DEG and DM enrichment signaling pathways on 32 wk; (**D**) top 10 pathways of the intersection of DEG and DM enrichment signaling pathways on 32 wk.

To further investigate the biological function and regulatory mechanism underlying the different light intensities, we conducted a combined analysis of the signaling pathways enriched in both DEGs and DMs. The results revealed that there were 20 signaling pathways commonly enriched in both DEGs and DMs between NL group and LL group on 22 wk (Figure 7A,B; Table S12). The top 10 shared pathways of the largest number of DEGs and DRMs were neuroactive ligand–receptor interaction, steroid hormone biosynthesis, the biosynthesis of amino acids, pyrimidine metabolism, folate biosynthesis, glycerophospholipid metabolism, nicotinate and nicotinamide metabolism, ferroptosis, necroptosis and fructose and mannose metabolism.

At 32 wk, there were 25 signaling pathways commonly enriched in both DEGs and DMs between the NL group and LL group (Figure 7C,D; Table S13). The top 10 shared pathways of the largest number of DEGs and DRMs were the insulin signaling pathway, steroid hormone biosynthesis, pyrimidine metabolism, drug metabolism-other enzymes, the metabolism of xenobiotics by cytochrome P450, carbon metabolism, the FoxO signaling pathway, glycolysis/gluconeogenesis, the biosynthesis of amino acids and porphyrin and chlorophyll metabolism.

## 3. Discussion

Light is an essential environmental factor in the regulation of avian reproductive activity [6]. In the present study, in order to investigate the effect of light intensity on the ovarian development and reproductive performance of Partridge chicken, we gave the broiler breeders a weak-light-intensity treatment. Our results showed that although the LL group had slower ovarian development and a later age at first egg than the NL group, the overall laying performance was better than that of the NL group. We found that on 32 wk, in the NL group, the number of LYFs was significantly lower and the number of SYFs was significantly higher than in the LL group. The laying performance of the chicken depends on ovarian development and well-organized follicular hierarchy. During egg laying in female birds, dominant follicles are selected from the small growing follicles (such as SYFs or WFs) pool, then develop into pre-ovulatory follicles (LYFs) for ovulation and egg production. Those unselected follicles most often undergo atresia [19]. The accumulation of SYF causes the disorganization of follicular development, increasing the loss of follicles and thus shortening the egg-laying period. In a previous study, it was also found that follicular development was disorganized, leading to a decrease in egg production performance in broiler breeders [19,20]. Therefore, in the present study, low-light-intensity treatment inhibited the overgrowth of follicles and maintained their hierarchical structure, thereby maximizing their egg-laying performance and prolonging the laying period in LL group.

Ovarian development is regulated by reproductive hormones. However, our results were different compared to our expectations. The results showed that reproductive hormone levels were higher in the LL group than in the NL group on 22 wk. Previous studies have shown that broiler breeders will also undergo laying when exposed to non-stimulating light conditions for a long time (red light rearing or a short photoperiod) [21,22,23]. This suggested that exposure to low light intensity for a long time may cause photo-adaptation, which subsequently initiates ovarian development by up-regulating the reproductive hormone levels.

To further investigate the molecular mechanism of the light-intensity regulation of ovarian development, we used transcriptomics to understand the gene regulation involved in this process and to identify key regulators and signaling pathways. Our results showed that a total of 128 and 155 DEGs were identified in the NL group and LL group during the pre-laying period (22 wk) and laying period (32 wk), respectively. It is worth noting that many of these DEGs have been identified as key regulators in the process of ovarian development. For example, at 22 wk, we found the up-regulation of cytochrome P450 family 21 subfamily A member 2 (*CYP21A1*) and the down-regulation of somatostatin receptor 2 (*SSTR2*) and steroid 5 alpha-reductase 2 (*SRD5A2*) in the LL group. Those genes were enriched in steroid hormone biosynthesis and the neuroactive ligand–receptor interaction pathway. Studies have demonstrated that *CYP21A1* is a key factor that promotes the secretion of reproductive hormones, and the overexpression of *CYP21A1* can promote the proliferation of granulosa cells, thereby promoting ovarian development in chickens [24]. In an in vitro study of cultured bovine granulosa cells, si-*SSTR2* was found to significantly increase P4 levels and the expression of key factors for steroid hormone synthesis [25]. This result further validated our previous speculation that the LL group had initiated ovarian development at 22 wk. We found that neuropeptide Y (*NPY*) was enriched in the neuroactive ligand–receptor interaction pathway at 32 wk. It was shown that *NPY* is involved in the regulation of LH secretion, which has a facilitating effect on promoting granule cell proliferation [26,27,28,29]. DEGs were also enriched in cytokine–cytokine receptor interaction, as well as the TGF-beta signaling and MAPK signaling pathways. These pathways have been found to regulate the involvement in the follicular development process and affect avian egg-laying traits [30,31,32,33]. These results suggest that the prolongation of the laying period in the LL group might be regulated by these pathways.

We used metabolomics to further explore the key metabolites and metabolic pathways associated with the light-intensity regulation of ovarian development, and analyzed the screened DMs for KEGG enrichment. Those DMs were mostly enriched in glycerophospholipid metabolism, steroid hormone biosynthesis and ABC transporters. Previous studies have found that dietary energy has an important regulatory role in gonadal development and estrogen secretion in birds [34,35,36]. Restricted feeding trials with hens have shown that a slowdown in growth delayed the age at egg laying [37]. In addition, in avian species, lipids, especially triglycerides, are involved in the sexual maturation of hens [38]. Therefore, in the present study, the later age at 5% laying rate in the LL group may be due to the low-light-intensity treatment affecting the energy metabolism of the organism, slowing down the increase in body weight and down-regulating lipid metabolism in the ovary. The results at 32 wk showed that DMs were mainly enriched in phenylalanine metabolism, bile secretion, arginine and proline metabolism and steroid hormone biosynthesis. It was found that phenylalanine can regulate reproductive hormone secretion and the ovulation cycle in chickens through serotonin [39,40]. The GWAS study identified total bile acid as one of the metabolites associated with duck egg-laying candidate loci [41]. These results suggested that the prolongation of the laying period by low-light-intensity treatment may be achieved by these metabolites through the involvement and regulation of steroid hormone secretion. Integrated transcriptomics and metabolomics analyses showed that DEGs and DMs were enriched in both neuroactive ligand–receptor interaction and steroid hormone biosynthesis. It is worth noting that the DM involved in the neuroactive ligand–receptor interaction pathway was tryptamine, which easily induced the release of serotonin and dopamine, and it was found that serotonin had an inhibitory effect on the feeding behavior of avians, and the secretion of hormones in granulosa cells [42,43]. Furthermore, these DEGs and DMs were enriched in steroid hormone biosynthesis in both periods, suggesting that this pathway is a potential signaling pathway for light-intensity regulation of ovarian development.

In summary, low-light-intensity treatment delayed the laying time, slowed down the overdevelopment of follicles in the ovary, and prolonged the laying period in broiler breeders. Transcriptomic and metabolomic analyses showed that low light intensity regulated ovarian development through signaling pathways such as steroid hormone biosynthesis and neuroactive ligand–receptor interaction. In these pathways, *CYP21A1*, *SSTR2*, and *NPY* may be involved in regulating the synthesis of metabolites, including tryptamine, triglycerides and phenylalanine, which ultimately regulate ovarian development.

## 4. Materials and Methods

### 4.1. Animals and Experimental Design

Two hundred 5-week-old (5 wk) partridge broiler breeders with uniform body weights (668.15 ± 1.78 g) were selected for the experiment (Guangzhou Kwangfeng Industrial Co., Ltd. Guangzhou, China), and randomly divided into a low-light-intensity group (LL group) and a natural-light-intensity group (NL group) (*n* = 100, each group had 3 replicates). Both of them were in the semi-open chicken houses. Based on this system, in the LL group, we used a black cloth and shaded the chicken house to keep the chickens in low-light-intensity conditions. The NL group was not shaded, and reared in a natural light intensity. During the experiment, the photoperiodic system followed the feeding system of Guangzhou Kwangfeng Industrial Co., Ltd. (Guangzhou, China). In brief, during the rearing period, broiler breeders were exposed to an 8 h light/16 h dark cycle (8 L:16 D) from the growing period to the pre-laying period (5 wk to 22 wk). Then, 2 h light per week was added from 23 wk until 16 L:8 D (Figure 1B). Ovarian and blood samples were collected on 22 wk and 32 wk. Light intensity data were collected during the experiment. The light intensity of the LL group and NL group from 5 wk to 22 wk were 0.41 ± 0.05 lux and 45.39 ± 1.09 lux, respectively; from 23 wk to 32 wk they were 23.92 ± 0.06 lux and 66.93 ± 0.76 lux, respectively.

### 4.2. Samples Collection

The number of eggs laid in each group was recorded during the laying period for calculating the laying rate [laying rate (%) = total number of eggs laid during the statistical period/cumulative number of chickens on actual rearing day × 100%]. Blood samples from the wing vein were collected at 22 wk (pre-laying period) and 32 wk (laying period) to measure the reproductive hormone levels (*n* = 20). Tissue samples were collected after the chickens were stunned and decapitated between the cervical vertebrae and the base of the skull by following the sampling procedure of institutional animal care and use guidelines. Large yellow follicles (LYFs, diameter > 8 mm), small yellow follicles (SYFs, diameter = 6–8 mm) and white follicles (WFs, diameter < 6 mm) were counted [44], and the gonadosomatic index was calculated (GSI% = gonadal weight/body weight × 100%) (*n* = 10). Ovary samples were collected from each group and immediately frozen in liquid nitrogen until homogenization for transcriptomics and metabolomics analysis (*n* = 6).

### 4.3. Detection of Serum Reproductive Hormones

Serum was separated from the blood samples by centrifugation at 3500 rpm at 4 °C for 15 min, and used for reproductive hormone measurement. The hormone levels of Estradiol (E2), progesterone (P4), luteinizing hormone (LH) and follicle-stimulating hormone (FSH) were measured by ELISA method. The assay sensitivity of the E2, P4, LH and FSH kits (Enzyme-linked Biotechnology, Shanghai, China)were 1.0 pg/mL, 0.1 ng/mL, 0.5 mIU/mL and 0.1 mIU/mL, respectively. The intra- and inter-assay variation coefficients were below 15%, and the r values of the assay standard curves were >0.99.

### 4.4. Ovarian RNA Extraction and Sequencing

Total RNA was isolated from the ovary samples (*n* = 6) using TRIzol^®^ Reagent (Life Technologies, CA, USA). RNA samples were analyzed based on the A260/A280 absorbance ratio using a Nanodrop ND-2000 system (Thermo Fisher Scientific, Wilmington, DE, USA). The integrity of RNA was evaluated using the RNA Nano 6000 Assay Kit of the Agilent Bioanalyzer 2100 system (Agilent Technologies, Santa Clara, CA, USA).

Paired-end libraries were prepared using a Hieff NGS Ultima Dual-mode mRNA Library Prep Kit [Yeasen Biotechnology (Shanghai) Co., Ltd., Shanghai, China] following the manufacturer’s instructions, and the libraries’ quality was evaluated with an Agilent Bioanalyzer 2100 system. The libraries were sequenced on an Illumina NovaSeq platform to generate 150 bp paired-end reads, which were further processed with a bioinformatic pipeline tool, the BMKCloud (www.biocloud.net, accessed on 31 August 2023) online platform. Raw data (raw reads) of the fastq format were filtered using in-house Perl scripts to remove reads containing adapters and ploy-N sequences, and low-quality reads. The clean data were calculated for Q20, Q30, GC-content and sequence duplication level. Clean reads were mapped to the reference genome (*Gallus_gallus*. GRCg6a.genome. fa) using Hisat2 v2.0.4 [45]. Subsequently, StringTie (version 2.2.1) was used to assemble transcripts after integrating all individual transcripts and quantitatively expressed genes [46]. The Fragments Per Kilobase of transcript per Million mapped reads (FPKM) of each gene were calculated based on the gene length and the corresponding mapped read counts. Genes were considered differentially expressed (DEGs) with *p* < 0.05 and |log2 fold change| > 1 using the DESeq2 R package (1.16.1) [47].

### 4.5. Liquid Chromatograph-Mass Spectrometer Analysis

An untargeted metabolomics approach was used to detect metabolite changes. The extraction, detection, and quantitative analysis of metabolites in ovarian samples were performed by BioMarker Biotechnology Co., Ltd. (Wuhan, China).

The raw data were collected by MassLynx V4.2, processed by Progenesis QI software v2.0, and identified by Progenesis QI software v2.0 online METLIN database and Biomark’s self-built library. Theoretical fragment identification and mass deviation were within 100 ppm. After being normalized to total peak intensity, the processed data were subjected to multivariate data analysis by an R package (ropls v1.6.2), including principal component analysis (PCA) and orthogonal partial least-squares (OPLS-DA). OPLS-DA was used to perform permutation tests 200 times to verify the reliability. The variable importance in the projection (VIP) value of the model was calculated using multiple cross-validation. The method of combining the difference multiple, the *p* value and the VIP value of the OPLS-DA model was adopted to screen the differential metabolites (DMs). The screening criteria are FC > 1, *p* value < 0.05 and VIP > 1.

### 4.6. Functional and Enrichment Analysis

The cluster profiler R software package (ropls v1.6.2) was used for Gene Ontology (GO) function enrichment and Kyoto Encyclopedia of Genes and Genomes (KEGG) pathway enrichment analyses [48].

### 4.7. Statistical Analysis

GraphpadPrism 8 and SPSS 26.0 were used for statistical analysis and the graphing of the data. The data were presented as mean ± SEM. Shapiro-Wilk test was used to analyze the normal distribution. Student *t*-test was used to analyze the differences between the LL group and the NL group. Data were presented as mean ± SEM. *p* < 0.05 indicates significant differences, and *p* > 0.05 indicates no significant differences.

## Figures and Tables

**Table 1:** Effects of different light intensities on laying performance.

Indicators	LL Group	NL Group	*p* Value
Age at 5% laying rate (d)	165.75 ± 0.75	160.50 ± 0.34	0.002
ALP ^1^ (wk)	32.25 ± 0.75	29.17 ± 0.17	0.023
Total laying rate ^2^ (%)	62.3 ± 0.01	60.21 ± 0.02	0.437
Total days of laying rate > 70%	63.75 ± 0.95	57.67 ± 1.50	0.009

^1^ ALP, age at laying peak; ^2^ Laying rate (%) = number of eggs laid/number of chickens × 100%.

**Table 2:** Effects of different light intensities on serum reproductive hormone levels at 22 wk.

Indicators	LL Group	NL Group	*p* Value
P4 (ng/mL)	18.97 ± 0.73	16.44 ± 0.74	0.019
E2 (pg/mL)	310.56 ± 17.62	271.34 ± 17.26	0.120
LH (mIU/mL)	13.99 ± 0.40	12.39 ± 0.53	0.022
FSH (mIU/mL)	9.67 ± 0.69	9.21 ± 0.55	0.604

Data are presented as the mean ± SEM, *n* = 20.

**Table 3:** Effects of different light intensities on serum reproductive hormone levels at 32 wk.

Indicators	LL Group	NL Group	*p* Value
P4 (ng/mL)	17.01± 0.57	20.63 ± 0.49	<0.0001
E2 (pg/mL)	309.40 ± 10.50	399.76 ± 8.57	<0.0001
LH (mIU/mL)	13.88 ± 0.55	16.94 ± 0.37	<0.0001
FSH (mIU/mL)	9.10 ± 0.72	12.36 ± 0.42	0.0005

Data are presented as the mean ± SEM, *n* = 20.

## Data Availability

The raw sequence data in this paper have been deposited in the China National GeneBank DataBase (CNGBdb) under the accession number PRJCA027959. Any remaining data could be acquired in the supplementary files or through a request to the corresponding author.

## Supplementary Materials

The following supporting information can be downloaded at: https://www.mdpi.com/article/10.3390/ijms25168704/s1.
